# When Do Natural Language Metaphors Influence Reasoning? A Follow-Up Study to Thibodeau and Boroditsky (2013)

**DOI:** 10.1371/journal.pone.0113536

**Published:** 2014-12-09

**Authors:** Gerard J. Steen, W. Gudrun Reijnierse, Christian Burgers

**Affiliations:** 1 Department of Language and Communication, VU University Amsterdam, Amsterdam, The Netherlands; 2 Department of Communication Science, VU University Amsterdam, Amsterdam, The Netherlands; Cardiff University, United Kingdom

## Abstract

In this article, we offer a critical view of Thibodeau and Boroditsky who report an effect of metaphorical framing on readers' preference for political measures after exposure to a short text on the increase of crime in a fictitious town: when crime was metaphorically presented as a beast, readers became more enforcement-oriented than when crime was metaphorically framed as a virus. We argue that the design of the study has left room for alternative explanations. We report four experiments comprising a follow-up study, remedying several shortcomings in the original design while collecting more encompassing sets of data. Our experiments include three additions to the original studies: (1) a non-metaphorical control condition, which is contrasted to the two metaphorical framing conditions used by Thibodeau and Boroditsky, (2) text versions that do not have the other, potentially supporting metaphors of the original stimulus texts, (3) a pre-exposure measure of political preference (Experiments 1–2). We do not find a metaphorical framing effect but instead show that there is another process at play across the board which presumably has to do with simple exposure to textual information. Reading about crime increases people's preference for enforcement irrespective of metaphorical frame or metaphorical support of the frame. These findings suggest the existence of boundary conditions under which metaphors can have differential effects on reasoning. Thus, our four experiments provide converging evidence raising questions about when metaphors do and do not influence reasoning.

## Introduction

The idea that metaphor can guide our thought has been researched in various new and exciting ways since the late 1970s [2]. In recent years, prominent metaphor scholars like George Lakoff [3] have argued that metaphors can even act as conceptual frames in important areas like politics, thereby offering distinct conceptual perspectives on all sorts of topics like political leaders, parties, elections, and other political events and issues, presumably affecting people's attitudes, intentions and actions [4]–[6]. However, these are claims about metaphor in thought that are largely based on language analysis [7], which has led to a call for empirical evidence based on reader-response data [8]. In two particularly prominent sets of studies, Thibodeau and Boroditsky [1], [9] have therefore experimentally investigated whether framing a text about crime with two different metaphors led to different political views and policy preferences of readers. They report consistent framing effects and conclude that metaphors do indeed influence reasoning.

In this article, we offer a critical view of Thibodeau and Boroditsky [1] and argue that their design has left room for alternative explanations. We report four experiments comprising a follow-up study. Our results reveal no metaphorical framing effect, but instead show that another process is at play. We conclude that the metaphorical framing effect reported by Thibodeau and Boroditsky [1], [9] as well as our own alternative explanation are findings that need more research.

### Reported metaphorical framing effects

In two sets of studies, Thibodeau and Boroditsky [1], [9] investigated the effects of metaphorical framing on thought. In Experiment 1 of the first series of studies [9], participants received two versions of a text about crime in the fictitious US city of Addison, one opening with the sentence “Crime is a wild beast preying on the city of Addison” while the other started with “Crime is a virus infecting the city of Addison.” Participants were then asked open questions about the policy measures that were needed to reduce crime in Addison. Responses showed that participants favored enforcement measures overall, but more so when they had seen the beast frame than when they had seen the virus frame. From this finding, Thibodeau and Boroditsky [9] conclude that the metaphorical frame (beast vs. virus) influences reasoning about a crime problem and its solutions.

These findings were subsequently replicated in a series of follow-up experiments. In a second experiment, the presentation of the metaphorical frame was reduced to a single opening phrase with only one metaphorically used word, “beast” or “virus”. The text looks like this:

Crime is a **beast/virus** ravaging the city of Addison. Five years ago Addison was in good shape, with no obvious vulnerabilities. Unfortunately, in the past five years the city's defense systems have weakened, and the city has succumbed to crime. Today, there are more than 55,000 criminal incidents a year - up by more than 10,000 per year. There is a worry that if the city does not regain its strength soon, even more serious problems may start to develop [9, p. 3].

The results again showed an overall tendency to favor enforcement measures but more so for the beast-framed text than for the virus-framed text. A third experiment ruled out the possibility that the framing effect could be attributed to lexical priming and concluded instead that it had to be due to the use of the initial metaphorical utterance as a frame for the rest of the text. A fourth experiment then extended the area of investigation to see whether people might be able to overcome the effect of the frame and prefer other options when these were presented as part of a list of alternatives. This time participants were asked to answer which area of possible counter-measures they thought should be examined in order to reduce crime. The results of this experiment, too, showed a greater preference for enforcement measures after reading “Crime is a beast” than after reading “Crime is a virus”. In the last experiment of the paper, Thibodeau and Boroditsky [9] moved the metaphorical frame to the last position of the text, attempting to test whether metaphorical frames work by assimilation of metaphorical information during reading of the complete text (as in the previous versions) or by activating a fossilized package of pre-existing ideas when it occurs in final position (in the new versions for this particular experiment). This time, metaphorical frames did not have an effect on people's preference for which particular aspect of crime should be examined in order to reduce crime. The authors conclude that “metaphors can gain power by coercing further incoming information to fit with the relational structure suggested by the metaphor” ([9], p. 3).

In a second series of studies Thibodeau and Boroditsky [1] pursued this line of enquiry by focusing on the specific solutions people are ready to adopt on the basis of this reasoning. In three experiments using the same text as above, they studied whether metaphorical frames could influence readers' preference for adopting a particular crime policy measure. After reading, participants were asked to indicate which of the following five measures they considered best for the situation described in the stimulus text, with some variation between tasks across the three experiments:

1. Increase street patrols that look for criminals.
2. Increase prison sentences for convicted offenders.
3. Reform education practices and create after school programs.
4. Expand economic welfare programs and create jobs.
5. Develop neighborhood watch programs and do more community outreach.

Their findings demonstrate that participants who were exposed to the beast frame chose more enforcement-oriented measures (i.e., policies 1, 2 and 5) than participants exposed to the virus frame. This is in line with the conclusions drawn before: “metaphors influenced people's reasoning even when they had a set of options available to compare and select among” ([1], p. 1).

An additional interest in these studies was the role of metaphor awareness. In a post-exposure question, people were always asked whether they could remember the framing word (“beast” or “virus”) in a cloze test for the initial sentence. Only few participants were able to remember the metaphor, and these data did not influence the policy preference findings. As a result of these findings, Thibodeau and Boroditsky [1] conclude that natural language metaphors covertly influence reasoning.

### Problems and hypotheses

There are several questions that can be raised about these studies. The first has to do with the authors' claim that “Even with this minimal one-word metaphorical intervention, we found that participants offered different problem solving suggestions, consistent with the metaphors” ([1], p. 2). Consider the following highlighted words in the stimulus text:

Crime is a beast/virus **ravaging** the city of Addison. Five years ago Addison was **in good shape**, with no **obvious vulnerabilities**. Unfortunately, in the past five years the city's **defense systems have weakened**, and the city has **succumbed to** crime. Today, there are more than 55,000 criminal incidents a year - up by more than 10,000 per year. There is a worry that if the city does not **regain its strength** soon, even more serious problems may start to develop.

We argue that all bold words can be read as metaphors that either continue the beast or the virus frame. Thus, when readers arrive at “in good shape” and “no obvious vulnerabilities”, they may connect these phrases with the virus and beast referents in their situation model arising from the first sentence, respectively, extending the metaphorical frame of beast or virus to the second sentence. The same holds for “defense systems have weakened” in the next sentence, which may be seen as compatible with both the virus and the beast frame. Whether “regain its strength” in the last sentence may still be attached to both of these continued frames is a moot point, as the persistence of the frame has been interrupted by the fourth sentence that only focuses on the target domain of crime. Thus, the text following the beast/virus metaphor can be analyzed as supporting two alternative interpretations that each maintain and develop the initial metaphorical frame by a number of further expressions, potentially keeping it alive and elaborating it in two different directions by means of a series of metaphorical structures from beginning to end.

Even though this effect of potential metaphorical support is acknowledged and even included in the argumentation of Thibodeau and Boroditsky [1], [9], it is not clear what language or text mechanism their study eventually engages with. One aim of the present study is therefore to separate the potential effect of the metaphor at the beginning of the text from the other metaphors that potentially support it in the following sentences. We study whether placing a metaphorical frame like “Crime is a beast” or “Crime is a virus” always influences reasoning or only in special circumstances (when the metaphorical frame is supported by other metaphors). To this end, an alternative version of the Thibodeau and Boroditsky text was designed that contained no supporting metaphors (see Table 1). If it is true that it is just the one-word metaphorical frame at the beginning of the text that causes the effect on policy preference, then the revised text without supporting metaphors in the following sentences should perform equally well as the original text that has the series of supporting metaphors. However, there is the alternative possibility that the supporting metaphors do act as such prompts, for the reason that they have been used as metaphorical elaborations of the one word initial metaphorical frame. This would in fact make the metaphorical elaborations responsible for the overall framing effect of the initial one-word metaphorical manipulation, since they involve an extended expression of the metaphorical frame.

**Table 1:** Metaphorically framed texts with and without additional metaphorical support.

Original text, with metaphorical support	Alternative version, without additional metaphoric support
(1) Crime is a beast/virus/problem **ravaging** the city of *Almere/Addison.*	(1) Crime is a beast/virus/problem **with disastrous effects on** the city of *Almere/Addison.*
(2) Five years ago *Almere/Addison* was **in good shape**, with no obvious **vulnerabilities**.	(2) Five years ago *Almere/Addison* was **secure**, with no **risks of decline**.
(3) Unfortunately, in the past five years the city's **defense systems have weakened**, and the city has **succumbed to** crime.	(3) Unfortunately, in the past five years the city's **measures to maintain security have been less successful**, and crime has **increased at an alarming rate**.
(4) Today, there are more than *3,200/55,000* criminal incidents a year - up by more than *600/10,000* per year.	(4) Today, there are more than *3,200/55,000* criminal incidents a year - up by more than *600/10,000* per year.
(5) There is a worry that if the city does not **regain its strength soon**, even more serious problems may start to develop.	(5) There is a worry that if the city does not **improve its policies soon**, even more serious problems may start to develop.

Note. Underlined words indicate the framing manipulation. Participants were exposed to one of the two metaphors (beast, virus) or the non-metaphoric control condition (problem). Words in boldface indicate elements with or without metaphorical support. Words in italics indicate differences between Experiment 1 (Netherlands) and Experiments 2–4 (USA): We set the Dutch experiment in a Dutch city (Almere). Because this Dutch city has little less than 200,000 inhabitants, we also adjusted the crime figures to fit a city of this size. In the US experiments, we again set the text in the fictitious city of Addison and used the crime figures mentioned in the stimulus text from Thibodeau and Boroditsky [1].

Our first hypothesis, therefore, is that the original text versions used by Thibodeau and Boroditsky [1], [9] show a framing effect on the basis of the elaboration of the initial metaphor “Crime is a beast/virus” in the rest of the text, which may disappear when the elaboration is removed and there is just the one-word metaphorical frame. We hence predict that the text versions without additional metaphorical support display a weaker or no framing effect compared to the text versions with additional metaphorical support.

With all of these considerations, a second issue about the Thibodeau and Boroditsky [1] study became apparent as well. Their studies did not compare the diverging effects of the metaphorically framed texts with the effect of a non-metaphorically framed text. Since the beast frame seems to increase a policy preference for enforcement relative to the virus frame, it is important to study how both frames fare compared to a neutral, non-metaphorical control. Furthermore, because both frames are valenced in favor of either enforcement (beast) or reform (virus), we expect that a neutral, non-metaphorical frame presenting crime as a problem ought to allow equally for enforcement-oriented and reform-oriented preferences. Our second hypothesis therefore holds that the effects of both the beast and the virus frame conditions ought to differ from a non-metaphorical framing condition (“Crime is a problem”) in which participants display neither a preference for an enforcement nor a preference for a social reform policy but, having an equal chance of preferring either type of measure, should be more neutral. These differences between metaphorical versus non-metaphorical frames should be stronger for the text version with metaphorical support than for the text version without metaphorical support.

Thirdly, the argument about metaphorical framing effects suggests a difference in policy preference that is caused by the metaphorically framed text. To further our insights into metaphorical framing, it is crucial to know whether the difference is relative to the other frame or whether participants have also shifted their prior beliefs in the direction of the metaphorical frame. However, Thibodeau and Boroditsky [1], [9] did not include a pre-treatment measure of policy preferences that can be used as a basis for comparison. With the overall goal of determining the magnitude of the metaphorical framing effect by reading, we extended the design of the study by another factor, including both pre- and post-reading measurements of policy preference. Our third hypothesis is therefore that exposure to the beast frame text should sway people's initial position more towards enforcement, exposure to the virus frame text should sway it more towards reform, and exposure to the neutral frame text should sit in between these two tendencies. There hence ought to be an interaction between pre-and post-exposure attitudes on the one hand and frames on the other. Again, these effects should be stronger for the text version with metaphorical support than for the text version without metaphorical support, which predicts a three-way interaction effect.

In sum, the present study examines the effect of metaphorical frames on policy preference by comparing the difference between policy preferences before and after exposure to the crime text. We test whether, after reading, the one-word virus frame increases people's preference for reform options, the one-word beast frame increases people's preference for enforcement options, and the problem frame occupies a middle position between the two preferences. In testing these predictions, the study also examines the interaction of these tendencies with support by other metaphors in the rest of the text, checking whether the predicted metaphorical framing effects are affected by additional metaphorical support in the rest of the text or not.

## Methods

### Ethics statement

Data were collected in accordance with ethical guidelines of our institution (see http://fsw.vu.nl/en/departments/communication-science/research/good-research-practice-guidelines/index.asp). The study was approved by the Ethical Committee of the Faculty of Arts of VU University Amsterdam. Participants volunteered to partake in the study of their own free will. Their informed consent was recorded by their self-chosen continuation with the study after they had read a notification on the first page of the survey that their data would be processed anonymously, that they could quit the study at any given time without stating their reasons for doing so, and that by participating, they voluntarily granted us permission to use their data for the purpose of our research project.

### Design

#### Experiments 1 and 2

Experiments 1 and 2 were both extensions of the studies reported in Thibodeau and Boroditsky [1]. Both experiments employed a 3 (metaphorical frame: beast metaphor, virus metaphor, no metaphor) ×2 (metaphorical support: present, absent) ×2 (exposure: pre or post) mixed experimental design with exposure as a within-subjects factor and metaphorical frame and metaphorical support as between-subjects factors.

The main difference between Experiments 1 and 2 is that Experiment 1 was conducted in Dutch in the Netherlands and Experiment 2 in English in the US. We replicated Experiment 1 in the US, because various studies have demonstrated that the use and evaluation of specific metaphors can vary across cultures [10]–[12] and languages [13]–[14]. Another difference between Experiments 1 and 2 was that we used an existing Dutch town, Almere, in Experiment 1, as opposed to the original fictitious city Addison in the Thibodeau and Boroditsky studies. Our reasoning was that the Netherlands is too small to allow for the use of a fictitious town with such spectacular crime growth without people knowing about it, which might detract from the veridity of the experimental texts. Since the use of an existing town might influence our participants' views and subsequent judgments, however, we reverted to the fictitious city of Addison in Experiment 2. In sum, to rule out language and the reference to an actual city as potential alternative explanations of our findings in the first Dutch language study, we ran our experiment in the US with the fictitious city of Addison (Experiments 2, 3, and 4).

#### Experiments 3 and 4

A third potential alternative explanation for any differences in findings between Experiments 1–2 and the original experiments by Thibodeau and Boroditsky [1] could be that our newly inserted pre-test measures work as a prime, thereby influencing participant responses. To rule out this explanation, we ran the US study a second time while removing the pre-test measures from the survey in Experiment 3. This experiment thus had a 3 (metaphorical frame: beast metaphor, virus metaphor, no metaphor) ×2 (metaphorical support: present, absent) between-subjects experimental design.

A fourth potential alternative explanation for differences in findings between the original study [1] and our experiments could lie in a difference in the number of participants used in [1] compared to our three experiments. Therefore, we conducted a post-hoc power analysis using G*Power, version 3.1.9.2 [15]–[16]. We calculated the power for effects of metaphorical framing after exposure in Experiments 1–2 with the ANCOVA option in G*Power (taking pre-exposure as a covariate). We calculated the power for effects of metaphorical framing after exposure in Experiment 3 with the ANOVA option in G*Power. We set alpha at.05 for all power analyses. Given the design of Experiments 1, 2 and 3, the power to detect a medium-sized effect (*f* = .25, [17]) of metaphorical framing after exposure was.95 (critical *F*(2, 246)  = 3.03; Experiment 1),.96 (critical *F*(2, 252)  = 3.03; Experiment 2) and.95 (critical *F*(2, 246)  = 3.03, Experiment 3), respectively. However, the power to detect a small effect (*f* = 10, [17]) of metaphorical framing after exposure was only.27 (Experiment 1) and.28 (Experiments 2–3), respectively. This means that, based on our Experiments 1, 2, and 3, we cannot completely rule out the existence of a small effect of metaphorical framing. An *a priori* power analysis shows that we would have needed at least 967 participants per experiment to detect such a small effect with a power of.80. To investigate the possibility of obtaining a small effect of metaphorical framing, Experiment 4 had the same design and procedure as Experiment 3. However, in Experiment 4, we included a sufficient number of participants to be able to test for a small effect.

### Materials

For our experimental materials, we used the original stimulus texts of Thibodeau and Boroditsky [1] in which crime was metaphorically framed as either a beast or a virus. We added a version with a non-metaphorical frame (“Crime is a problem”) and a version without the metaphorical support from the original stimulus materials by Thibodeau and Boroditsky [1], thereby creating six versions of our stimulus text (see Table 1 for an overview).

There were also some small differences between stimulus texts in Experiment 1 compared to Experiments 2–4. In Experiment 1, we set the stimulus text in an actual Dutch city called Almere. Because Almere has fewer than 200,000 inhabitants, we changed the crime figures to match a city of this size. Furthermore, in Experiment 1, all materials were in Dutch, and Table 1 presents our English translation. In Experiments 2, 3 and 4, we again set the stimulus text in the fictitious US city of Addison and used the crime figures used in Thibodeau and Boroditsky [1]. In Experiments 2–4, all materials were in English.

### Instrumentation and Procedure

In all experiments, data were collected online through Qualtrics (www.qualtrics.com). Instrumentation and procedure were roughly equal across experiments, with small differences explained below.

After an opening page, participants in Experiments 1 and 2 were first asked to give their opinion about a set of five policy measures intended to reduce crime, asking them to rank order them by selecting the most effective one first. This page contained the five measures also used by Thibodeau and Boroditsky ([1], Experiments 3–4). They were presented as measures to reduce crime in the top 10 cities in the Netherlands (Experiment 1) or the US (Experiment 2). This was our pre-exposure policy preference measure.

In Experiment 1, participants were subsequently asked to rank order a set of six political issues in order of highest to lowest importance: (a) jobs, wages, welfare benefits, (b) hospitals, schools, universities, (c) foreigners, immigrants, asylum seekers, (d) religion, culture, art, (e) banks, the euro, the budget deficit, and (f) climate, environment, and nature. In Experiment 2, they were asked the same, but under point (e) the euro was replaced by the debt ceiling to make this point more relevant for the US situation.

Then we tapped a number of demographic variables. In Experiments 1–2, these were included after the question about ranking the political issues. In Experiments 3 and 4, these were included as the final questions in the survey. We first asked participants about their political affiliation. Because the Netherlands has a multi-party system, we asked participants in Experiment 1 to rank-order the six largest political parties in order of preference. These were *PvdA* (Labour Party), *SP* (Socialist Party), *CDA* (Christian Democrats), *D66* (Liberal Democrats), *VVD* (Conservative Party) and *PVV* (Freedom Party). We later recoded these into preference for left-wing parties (*PvdA* and *SP*), center parties (*CDA* and *D66*) and right-wing parties (*VVD* and *PVV*). In Experiments 2, 3 and 4, we tapped political affiliation by asking participants whether they identified themselves as Republicans, Democrats or Independents. Independents were subsequently asked whether they felt more conservative, more liberal, or middle. We also asked participants about their age, gender, nationality, their first language, level of education and the digits of their ZIP code.

Next we asked participants to read the experimental text, which was presented as a text from the web about crime in Almere (Experiment 1) or Addison (Experiments 2–4). Participants were randomly assigned to one of the six experimental conditions. Please note that, in Experiments 3 and 4, this was the first item in the questionnaire since no pre-exposure measure was included. Unknown to participants, a hidden timer recorded the number of seconds they spent on this page.

In Experiments 1–2, we asked participants after reading the text to list three keywords of the text they had just read. We used these keywords to filter out participants who had not read the text (and who filled in things like “don't know” or a random string of letters). Because this question was not included in the original experiments [1], it was not included in Experiments 3 and 4.

Participants were then asked to indicate their preferences for the same set of five policy measures that were presented earlier as intended to reduce crime in the top 10 cities in the Netherlands (Experiment 1) or the US (Experiment 2). This time, we asked participants to rank order the measures for effectiveness in reducing crime in Almere (Experiment 1) or Addison (Experiments 2–4), based on the text they had just read. This was our post-exposure policy preference measure. Please note that, for participants in Experiments 3 and 4, this was the first time they ranked these five measures.

Subsequently, a text box appeared asking participants to mention the aspect of the text that had influenced their judgment most. The final question in Experiments 1 and 2 then asked participants to fill out a blank in an incomplete sentence, which was the opening sentence of the text. The blank position was the slot for “problem”, “beast” or “virus”. In Experiments 3 and 4, this question was followed by the demographic questions mentioned above. No further items were measured. After the final question, participants were debriefed, informed that the stimulus text was fictional, thanked for their participation and provided with instructions to receive their remuneration.

### Participants

In all experiments, we collected and paid for our data through an online panel. Data were sampled either through a Dutch consumer database by a dedicated research company (Experiment 1) or through Amazon's Mechanical Turk (MTurk, www.mturk.com, Experiments 2–4). Participants were compensated with a small reward through the research company (Experiment 1) or with US$1 for participation (Experiments 2–4) through MTurk. Data were collected in April 2013 (Experiment 1), November 2013 (Experiment 2), December 2013 (Experiment 3) and August 2014 (Experiment 4).

Before data collection started, we set our sampling criteria. In Experiments 1–3, we aimed for 300 completed questionnaires (approx. 50 completed questionnaires per experimental condition). In Experiment 4, we aimed for 1,200 completed questionnaires (approx. 200 completed questionnaires per experimental condition). In Experiment 1, we aimed for an even distribution of participants across gender and three age groups (young: 18–34 years, middle: 35–54 years, old: 55+ years). When a specific quota was reached, participants of the specific gender or age group could no longer participate in the study. We also decided *a priori* that residents of Almere could not participate because the stimulus text was about the city of Almere, and filtered these out through their ZIP code. In Experiments 2, 3 and 4, all M-Turk participants (“Turkers”) had to have a high HIT Approval Rate of at least 80%, indicating that, on average, the worker completed at least 80% of tasks satisfactory. Turkers who had participated in Experiment 2 were excluded from participation in Experiment 3 and 4, and similarly, Turkers who had participated in Experiment 2 or 3 were excluded from participation in Experiment 4.

Before data analysis, we also decided to only include participants in our analyses who met a number of conditions. First, participants had to have either the Dutch (Experiment 1) or US nationality (Experiments 2–4), had to speak Dutch (Experiment 1) or English (Experiments 2–4) as their first language, and they had to be eligible to vote (i.e., 18 years or older). Based on these criteria, we excluded no participants from Experiment 1, but fourteen participants from Experiment 2, seven participants from Experiment 3 and 29 participants from Experiment 4. Participants who could not mention any keywords of the text were also deselected: 21 participants for Experiment 1 and two participants for Experiment 2. We also measured the time participants spent on the page with the stimulus text: participants who either read the text extremely quickly (i.e., under 5 seconds) or extremely slowly (i.e., more than 60 seconds) were removed from the data. Based on these criteria, we excluded 26 participants (Experiment 1), 26 participants (Experiment 2), 43 participants (Experiment 3) and 150 participants (Experiment 4). Including these participants did not alter the general pattern of results. We also checked and found that roughly the same number of participants were selected and de-selected in every experimental condition (Experiment 1: χ*^2^*(5)  = 4.76, *p* = .45; Experiment 2: χ*^2^*(5)  = 3.19, *p* = .67; Experiment 3: χ*^2^*(5)  = .91, *p* = .97; Experiment 4: χ*^2^*(5)  = 4.04, *p* = .54).

#### Experiment 1

A total of 772 volunteers were approached, 300 of whom completed the survey. Applying our exclusion criteria left 253 participants. Their average age was 45.85 years (*SD* = 15.73, range: 18–77 years). A total of 133 participants were female (52.6%). In terms of highest educational level, 6 participants had completed elementary school (2.4%), 84 participants had completed a form of high school (33.2%) and 163 participants (64.4%) had completed higher education. After recoding political party preference into left, middle and right, 96 participants (37.9%) described themselves as on the left, 68 participants (26.9%) as in the middle and 89 (35.2%) as on the right of the Dutch political spectrum.

#### Experiment 2

A total of 301 participants completed the survey. Applying our exclusion criteria left 259 participants. Their average age was 35.22 years (*SD* = 11.89, range: 18–72 years). A total of 125 (48.3%) participants were female. A total of 86 participants (33.2%) completed high school as their highest level of education while 127 participants (49.0%) and 46 participants (17.8%) completed an undergraduate or a graduate study, respectively. 52 (20.1%) participants described themselves as Republicans, 118 (45.6%) as Democrats and 89 (34.4%) as Independents. Of the participants who indicated that they were Independents, 16 participants (17.9%) considered themselves to be more conservative, 33 participants (37.1%) considered themselves to be more liberal, and 40 participants (44.9%) said they were in between.

#### Experiment 3

A total of 302 participants completed the questionnaire. Applying our exclusion criteria left 252 participants. Their average age was 35.04 years (*SD* = 12.03, range: 18–70 years). A total of 95 participants (37.7%) were female. Of all participants, 71 (28.2%) completed high school, 142 (56.3%) completed an undergraduate level of education, and the remaining 39 (15.5%) were graduates. A total of 38 participants (15.1%) indicated they were Republicans, 104 (41.3%) were Democrats, and 110 (43.7%) were Independents. Of the participants who identified themselves as Independents, 12 participants (10.9%) said they were more conservative, 47 (42.7%) said that they were more liberal, and 51 (46.4%) positioned themselves in the middle.

#### Experiment 4

A total of 1,205 participants completed the questionnaire. Applying our exclusion criteria left 1,026 participants. Their average age was 33.53 years (*SD* = 10.65, range: 18–79 years). A total of 438 participants (42.7%) were female. Of all participants, 1 (0.1%) completed elementary school, 5 (0.5%) completed middle school, 315 (30.7%) completed high school, 552 (53.8%) completed an undergraduate level of education, and the remaining 153 (14.9%) were graduates. A total of 169 participants (16.5%) indicated they were Republicans, 440 (42.9%) were Democrats, and 417 (40.6%) were Independents. Of the participants who identified themselves as Independents, 57 participants (13.7%) said they were more conservative, 185 (44.4%) said that they were more liberal, and 175 (42.0%) positioned themselves in the middle.

## Results

### Control analyses

Across experiments, we first established whether experimental conditions did not differ on any of the demographic variables.

#### Experiment 1

Participants were evenly distributed across experimental conditions regarding age (*F*(5, 247)  = 1.76, *p* = .12), education level (χ*^2^*(10)  = 9.54, *p* = .48) and political affiliation (χ*^2^*(10)  = 13.19, *p* = .21). We did find a gender difference across experimental conditions (χ *^2^*(5)  = 13.46, *p* = .02; Cramer's V = .23). Inspection of standardized residuals showed that there were relatively fewer men (*n = *10) and more women (*n* = 30) in the “beast” condition without additional metaphors, and that there were relatively more men (*n* = 28) and fewer women (*n* = 18) in the “virus” condition without additional metaphors. In order to control for effects of uneven sampling of participant gender on pre- and post-reading scores for policy preference, we examined the two-way relation between gender and exposure. There was no effect of gender on the difference between pre- and post-reading scores: (*F*(1, 251) <1). This alleviates the sampling problem noted above and prevents an undue influence of participant gender on the overall findings.

#### Experiment 2

Participants were evenly distributed across conditions regarding education level (*χ* ^2^(10)  = 11.72, *p* = .30), and political affiliation (*χ* ^2^(10)  = 4.21, *p* = .94). Regarding gender, the distribution showed a trend (*χ* ^2^(5)  = 11.02, *p* = .051, Cramer's V = .21). Inspection of the standardized residuals showed that there were more men (*n* = 27) and fewer women (*n* = 13) than expected in the control condition (“Crime is a problem”) without metaphorical support. There was no interaction effect between gender and exposure: (F(1, 257) <1). Gender did thus not affect our overall findings. Regarding age, the distribution was significantly different across conditions (F(5, 253)  = 3.32, *p*<.01, η_p_^2^ = .06): post-hoc tests with Bonferroni corrections showed that participants in the beast condition with metaphorical support were significantly older than participants in both virus conditions (with (*p*<.01) and without (*p*<.05) additional metaphors). Differences in the same direction for both control conditions (“Crime is a problem”, with and without metaphorical support after the initial frame) are a trend (with metaphorical support: *p* = .079, without metaphorical support *p* = .077). Further analyses showed that there was no interaction effect between age and exposure (F(1, 257)  = 1.08, *p* = .30). Age did thus not affect our overall findings.

#### Experiment 3

Participants were evenly distributed across experimental conditions regarding age (F(5, 246) <1), gender (*χ* ^2^(5)  = 9.29, *p* = .10), education level (*χ* ^2^(10)  = 6.17, *p* = .80), and political affiliation (*χ* ^2^(10)  = 6.63, *p* = .76). Because only 1 participant out of 252 completed middle school as their highest level of education, we collapsed this participant with those having completed high school as their highest level of education, to conduct a reliable statistical analysis.

#### Experiment 4

Participants were evenly distributed across experimental conditions regarding age (F(5, 1020) <1), gender (*χ* ^2^(5)  = 1.59, *p* = .90), education level (*χ* ^2^(10)  = 9.84, *p* = .45), and political affiliation (*χ* ^2^(10)  = 13.68, *p* = .19). Because only 6 participants out of 1,026 completed either elementary or middle school as their highest level of education, we collapsed these participants with those having completed high school as their highest level of education, to conduct a reliable statistical analysis.

### Hypothesis testing: Effects on reasoning

Policy preference scores were calculated for pre- and post-exposure measurements. We included the first two preferences for the five policy measures that were rank-ordered by participants, coding reform measures as 0 and enforcement measures as +1 (following [1], [9]). This yields a scale with three values: each participant either preferred two enforcement-oriented measures (+2), one enforcement-oriented and one reform-oriented measure (+1) or two reform-oriented measures (0). In our current analysis, then, a higher score represents a tendency towards enforcement while a lower score represents a tendency towards reform. The resulting mean policy preference scores, divided by moment of measurement (pre- versus post-reading), metaphorical frame (beast metaphor, virus metaphor, no metaphor), and metaphorical support (present, absent) are presented in Table 2.

**Table 2:** Experiments 1–4: Mean scores (and standard deviations) of preference for enforcement-oriented or reform-oriented policy measures as a factor of the metaphorical framing (problem, beast, virus), metaphorical support (present, absent) and, for Experiments 1–2, exposure (pre or post-reading of stimulus text), based on the top 2 of preferred choices.

	Experiment 1		Experiment 2		Experiment 3	Experiment 4
	Pre-reading	Post-reading	Pre-reading	Post-reading	Post-reading	Post-reading
**Crime is a Problem frame**						
Metaphorical support present	1.09 (.70)	1.38 (.68)	.77 (.81)	1.33 (.74)	1.25 (.69)	1.06 (.77)
Metaphorical support absent	1.21 (.75)	1.43 (.67)	.88 (.72)	1.20 (.65)	1.02 (.73)	1.14 (.74)
Overall	1.15 (.72)	1.40 (.67)	.83 (.76)	1.27 (.69)	1.13 (.72)	1.10 (.75)
**Crime is a Beast frame**						
Metaphorical support present	1.15 (.80)	1.43 (.72)	.85 (.78)	1.13 (.77)	1.05 (.71)	.99 (.69)
Metaphorical support absent	1.20 (.65)	1.48 (.64)	.66 (.68)	1.07 (.73)	1.12 (.76)	1.11 (.75)
Overall	1.18 (.73)	1.45 (.67)	.76 (.74)	1.10 (.75)	1.08 (.73)	1.05 (.75)
**Crime is a Virus frame**						
Metaphorical support present	1.30 (.61)	1.48 (.68)	.74 (.73)	1.24 (.76)	1.10 (.78)	1.11 (.72)
Metaphorical support absent	1.04 (.73)	1.44 (.72)	.64 (.64)	1.00 (.78)	1.11 (.78)	1.08 (.71)
Overall	1.16 (.68)	1.45 (.70)	.69 (.68)	1.11 (.78)	1.11 (.77)	1.09 (.72)
**Total**						
Metaphorical support present	1.18 (.71)	1.42 (.69)	.79 (.77)	1.23 (.76)	1.13 (.73)	1.05 (.73)
Metaphorical support absent	1.15 (.71)	1.45 (.67)	.72 (.68)	1.08 (.72)	1.08 (.75)	1.11 (.73)
Overall	1.16 (.71)	1.44 (.68)	.75 (.73)	1.15 (.74)	1.10 (.74)	1.08 (.73)

#### Experiment 1

Data were analyzed with a 3 (metaphorical frame: beast metaphor, virus metaphor, no metaphor) ×2 (metaphorical support: present, absent) ×2 (exposure: pre or post) mixed ANOVA with frame and metaphorical support as between-subjects variables, exposure as a within-subjects variable and policy preference as the dependent variable. We found a significant main effect of exposure to the crime text on policy preference (*F*(1, 247)  = 38.78, *p*<.001, η_p_^2^ = .14). Reading the crime text (regardless of experimental condition) makes participants shift their policy preferences more towards enforcement. We found no effects of frame (*F*(2, 247) <1), metaphorical support (*F*(1, 247) <1), interaction between frame and metaphorical support (*F*(2, 247) <1), interaction between frame and exposure (*F*(2, 247) <1), interaction between metaphorical support and exposure (*F*(1, 247) <1), or interaction between frame, metaphorical support and exposure (*F*(2, 247)  = 1.03, *p* = .36). Thus, the shift towards enforcement is the same for all participants, irrespective of metaphorical frame or metaphorical support.

#### Experiment 2

Data were analyzed in a similar way to Experiment 1. Again, we found an effect of exposure on preference for crime solutions (*F*(1, 253)  = 96.18, *p*<.001, η_p_^2^ = .28), indicating that after reading a text on crime, participants preferred enforcement-oriented options more than before reading the text. We found no main effects of frame (*F*(2, 253)  = 1.10, *p* = .34) or metaphorical support (*F*(1, 253)  = 1.59, *p* = .21). We also found no interaction effects between frame and metaphorical support (*F*(2, 253) <1), between frame and exposure (*F*(2, 253) <1), between metaphorical support and exposure (*F*(1, 253) <1), or between frame, metaphorical support and exposure (*F*(2, 253)  = 1.81, *p* = .17). These data confirm the general picture of Experiment 1 that reading a text about crime makes participants shift more towards enforcement-oriented solutions, regardless of metaphorical framing or metaphorical support.

#### Experiment 3

Data were analyzed with a 3 (metaphorical frame: beast metaphor, virus metaphor, no metaphor) ×2 (metaphorical support: present, absent) between-subjects ANOVA with policy preference as the dependent variable. We found no effect of frame (*F*(2, 246) <1), no effect of metaphorical support (*F*(1, 246) <1) and no interaction effect between metaphorical frame and metaphorical support: (*F*(2, 246) <1).

#### Experiment 4

Data were analyzed in a similar way to Experiment 3. We found no effect of frame (*F*(2, 1020) <1), no effect of metaphorical support (*F*(1, 1020)  = 1.59, *p* = .21) and no interaction effect between metaphorical frame and metaphorical support: (*F*(2, 1020)  = 1.01, *p* = .36).

In Experiments 1 and 4, we found that reading time was not related to our dependent variable of the top 2 of solutions (Experiment 1: *r* = −.096, *p* = .12; Experiment 4: *r* = .052, *p* = .095). In Experiments 2 and 3, we did find that the reading time measure was positively related to our dependent variable of the top 2 of solutions, indicating that participants who took longer to read the text were likelier to lean towards enforcement-oriented solutions (Experiment 2: *r* = .16, *p*<.01; Experiment 3: *r* = .23, *p*<.001). When we added reading time to the analysis as a covariate, the general pattern of results remained unchanged. That is, in both experiments, reading time was again positively related to the top 2 of solutions, indicating that participants who took longer to read the text were likelier to lean towards enforcement-oriented solutions. All other outcomes were similar to the ones described above (i.e., the analysis without reading time as a covariate).

### Alternative analyses (1): Top 1 of preferred solutions

In the analyses reported above, we consistently studied whether metaphoric framing affects policy preference based on the top 2 of solutions. One possible explanation for differences between our findings and those of Thibodeau and Boroditsky [1] could lie in the fact that the original study only analyzed the top 1 of solutions. In doing so, this top 1 of policy choice was treated as a nominal dependent variable in a binary logistic regression analysis.

To see if our analyses differ if we analyze our results in that way, we conducted a binary logistic regression on the top 1 of policy preferences. Table 3 shows the descriptive statistics. First, we ran these analyses for our data of Experiments 1 and 2 with the pre-test top 1 choice (i.e., policy choice before exposure to the news text), metaphorical frame (beast metaphor, virus metaphor, no metaphor), and metaphorical support (present, absent) as predictors in Block 1, the two-way interactions of these variables as predictors in Block 2 and the three-way interaction as a predictor in Block 3. In Experiment 1, our model parameters were as follows: *R^2^* = .14 (Cox & Snell),.21 (Nagelkerke). Model *χ* ^2^(4)  = 36.73, *p*<.001. In Experiment 2, our model parameters were as follows: *R^2^* = .24 (Cox & Snell),.32 (Nagelkerke). Model *χ* ^2^(4)  = 71.16, *p*<.001.

**Table 3:** Number of participants (and total N per condition) who showed preference for enforcement (over reform) solutions, as a factor of the metaphorical framing (problem, beast, virus), metaphorical support (present, absent) and, for Experiments 1–2, exposure (pre or post-reading of stimulus text).

	Experiment 1		Experiment 2		Experiment 3	Experiment 4
	Pre-reading	Post-reading	Pre-reading	Post-reading	Post-reading	Post-reading
**Crime is a Problem frame**						
Metaphorical support present	22 (45)	37 (45)	17 (39)	27 (39)	23 (36)	90 (162)
Metaphorical support absent	26 (42)	30 (42)	15 (40)	24 (40)	22 (44)	94 (167)
**Crime is a Beast frame**						
Metaphorical support present	23 (40)	30 (40)	17 (47)	27 (47)	27 (44)	92 (178)
Metaphorical support absent	23 (40)	33 (40)	11 (44)	19 (44)	25 (43)	97 (180)
**Crime is a Virus frame**						
Metaphorical support present	27 (40)	34 (40)	14 (42)	26 (42)	22 (40)	100 (172)
Metaphorical support absent	26 (46)	38 (46)	14 (47)	27 (47)	27 (45)	87 (167)

The analyses of **Experiments 1 and 2** showed a positive effect of pre-test preference, indicating that participants who preferred the enforcement-oriented solution prior to exposure also had a preference for the same policy after exposure (Experiment 1: *B* = 2.01, *SE* = .37, Exp(*B*)  = 7.46, *p*<.001; Experiment 2: *B* = 2.66, *SE* = .41, Exp(*B*)  = 14.22, *p*<.001). We found no effects of metaphorical support (Experiment 1: *B* = .19, *SE* = .31, *p* = .58; Experiment 2: *B* = .31, *SE* = .29, *p* = .28). Most importantly, metaphorical frame also did not affect policy preference (Experiment 1: *df* = 2, Wald  = .88, *p* = .64; beast metaphor: *B* = .08, *SE* = .40, *p* = .85; virus metaphor: *B* = .38, *SE* = .42, *p* = .37; Experiment 2: *df* = 2, Wald  = 2.54, *p* = .28; beast metaphor: *B* = −.50, *SE* = .36, *p* = .17; virus metaphor: *B* = −.04 *SE* = .36, *p* = .92). Including any two-way or three-way interaction effect did not significantly improve the model. These are thus not reported upon.

In the alternative analyses for **Experiments 3 and 4**, we ran a binary logistic regression analysis with metaphorical frame (beast metaphor, virus metaphor, no metaphor) and metaphorical support (present, absent) as predictors in Block 1, and the two-way interaction of these variables as a predictor in Block 2. In Experiment 3, our model parameters were as follows: *R^2^* = .002 (Cox & Snell),.003 (Nagelkerke). Model *χ* ^2^(3)  = .59, *p* = .90. In Experiment 4, our model parameters were as follows: *R^2^* = .001 (Cox & Snell),.001 (Nagelkerke). Model *χ* ^2^(3)  = .85, *p* = .84. This analysis showed no effect of metaphorical support (Experiment 3: *B* = .16, *SE* = .26, *p* = .54; Experiment 4: *B* = .04, *SE* = .13, *p* = .75). We also found no effect of metaphorical framing on policy preference (Experiment 3: *df* = 2, Wald  = .19, *p* = .91; beast metaphor: *B* = .14, *SE* = .32, *p* = .67; virus metaphor: *B* = .05, *SE* = .32, *p* = .86; Experiment 4: *df* = 2, Wald  = .75, *p* = .69; beast metaphor: *B* = −.13, *SE* = .15, *p* = .41; virus metaphor: *B* = −.03, *SE* = .16, *p* = .84). Including the interaction effect did not significantly improve the model. This effect is thus not reported upon.

Summarizing, Experiments 1 and 2 showed converging evidence indicating that participants' attitude prior to exposure explained their preferred policy solution after exposure. Furthermore, in all four experiments, we found no significant effects of metaphorical frame or metaphorical support on preference for policy solutions. These analyses confirm the general pattern established in our original analyses.

### Alternative analyses (2): Effects of memory

In their papers, Thibodeau and Boroditsky [1], [9] argue that their effects are found irrespective of whether participants remembered the metaphoric frames. To explore whether similar results could be found in our data, we explored the relations between metaphorical frame, memory for metaphorical frame and policy preference.

We coded the answers to the memory question in two different ways. First, we coded whether participants had correctly remembered the framing word (“beast*”*, “virus*”* or *“*problem”). Second, we coded whether participants had correctly remembered the framing concept. This variable broadened our view of memory for frame from correct remembrance of the actual framing words (“beast*”*, “virus*”* or *“*problem”) to memory for all plausibly related words (such as “monster”, or “disease”, or “issue”; ‘memory for framing concept').

#### Experiment 1

We first tested whether memory was affected by exposure, metaphorical frame or metaphorical support. First, a logistic regression showed that memory for each of the three framing words was not significantly influenced by pre-exposure policy preference (*B* = −.14, *SE* = .35, *p* = .65), metaphorical frame (Wald  = 1.43, *p* = .49), and metaphorical support (*B* = −.26, *SE* = .31, *p* = .40). Adding two-way or three-way interactions did not improve model fit. These are not reported upon.

Then we broadened our view of memory for frame from correct remembrance of the keywords in the text (“beast*”*, “virus*”* or *“*problem”) to words appearing in the memory data that can be plausibly related to these keywords as suggesting memory for the same concept (such as “monster”, or “disease”, or “issue”; ‘memory for framing concept’), which yielded a different result. Now, the interaction between metaphorical frame and metaphorical support was a significant predictor of memory for metaphorical framing concept (Wald  = 6.05, *p*<.05), pointing to the fact that the virus frame was remembered significantly better in the text with metaphorical support than in the text without metaphorical support (*B* = 1.83, *SE* = .75, *p*<.05) whereas the effect of the beast frame on memory did not differ in relation to the presence or absence of metaphorical support (*B* = .86, *SE* = .75, *p* = .25).

Then we conducted a 3 (metaphorical frame: beast, virus or no metaphor) ×2 (memory for framing word: correct, incorrect) ×2 (exposure: pre or post) mixed ANOVA, with metaphorical frame and memory for metaphorical framing word as between-subjects variables, exposure as a within-subjects variable, and policy preference as the dependent variable. As in our previous main analysis, we found a significant main effect on policy preference of exposure to the crime text (*F*(1, 247)  = 26.73, *p*<.001, η_p_^2^ = .10), but no effects of metaphorical frame (*F*(2, 247) <1), memory for metaphorical framing word (*F*(1, 247) <1), or their interaction (*F*(2, 247) <1). Again, the shift towards enforcement is the same for all participants, irrespective of metaphorical frame or their memory of that frame. Broadening our view of memory for framing word to memory for framing concept again yielded similar results.

#### Experiment 2

A logistic regression showed that memory for each of the three framing words was not significantly influenced by pre-exposure policy preference (*B* = −.74, *SE* = .64, *p* = .25), but this was different for metaphorical frame, metaphorical support, and the interaction between metaphorical frame and metaphorical support, which all displayed significant effects. In particular, both metaphorical frames were remembered worse compared to the non-metaphorical problem frame (beast: *B* = −2.60, *SE* = .62, *p*<.001; virus: *B* = −1.35, *SE* = .58, *p*<.05). Moreover, the interaction terms between metaphorical frame and metaphorical support showed that both the beast (*B* = 3.19, *SE* = .77, *p*<.001) and the virus (*B* = 1.40, *SE* = .68, *p*<.05) frames were better remembered in the text versions with metaphorical support than in the ones without metaphorical support. Metaphorical framing words were remembered worse in the text with metaphorical support than in the text without metaphorical support (*B* = −1.36, *SE* = .60, *p*<.05). The same analysis with memory for framing concepts yielded a slightly different result. The metaphorical beast frame was again remembered worse than the non-metaphorical problem frame (*B* = −2.55, *SE* = .63, *p*<.001), but this was not the case with the metaphorical virus frame (*B* = −.66, *SE* = .62, *p* = .28). The interaction between beast and metaphorical support was significant (*B* = 3.18, *SE* = .72, *p*<.001), exhibiting the same positive effect of metaphorical support for beast in relation to memory for metaphorical frame; the interaction effect of virus with metaphorical support was non-significant, however (*B* = 1.13, *SE* = .71, *p* = .11).

Then we conducted a 3 (metaphorical frame: beast, virus, no metaphor) ×2 (memory for frame: correct, incorrect) ×2 (exposure: pre or post) mixed ANOVA, with metaphorical frame and memory for metaphorical framing word as between-subjects variables, exposure as a within-subjects variable, and policy preference as the dependent variable. As in our previous main analysis, we found a significant main effect on policy preference of exposure to the crime text (*F*(1, 253)  = 86.14, *p*<.001, η_p_^2^ = .25), but no effects of metaphorical frame (*F*(2, 253) <1), memory for metaphorical framing word (*F*(1, 253) <1), or their interaction (*F*(2, 253) <1). Thus, the shift towards enforcement is the same for all participants, irrespective of their memory for frame. Broadening our view of memory for framing word to memory for framing concept again yielded similar results.

#### Experiment 3

A logistic regression showed that memory for each of the three framing words and concepts was significantly influenced by metaphorical frame (words: *df* = 2, Wald  = 9.39, *p*<.01; concepts: *df* = 2, Wald  = 14.65, *p* = .001) but not by metaphorical support (words: *B* = −.41, *SE* = .28, *p* = .14; concepts: *B* = −.36, *SE* = .30, *p* = .23). The beast frame was significantly less well remembered than the non-metaphorical problem frame (words: *B* = −1.04, *SE* = .35, *p*<.01; concepts: *B* = −.93, *SE* = .36, *p*<.01), whereas memory for the virus frame was comparable to the non-metaphorical problem frame (words: *B* = −.43, *SE* = .36, *p* = .24; concepts: *B* = .43, *SE* = .42, *p* = .31). Including the interaction did not improve model fit and is thus not reported upon.

Then we conducted a 3 (metaphorical frame: beast, virus, no metaphor) ×2 (memory for frame: correct, incorrect) ANOVA, with metaphorical frame and memory for metaphorical framing word as between-subjects variables and policy preference as the dependent variable. As in our previous main analysis, there were no effects of metaphorical frame (*F*(2, 246) <1), memory for metaphorical framing word (*F*(1, 246) <1), or their interaction (*F*(2, 246) <1). Broadening our view of memory for framing word to memory for framing concept again yielded similar results. Please note that, across our first three experiments, also including the ‘metaphorical support’ variable in this exploratory analysis led to highly uneven spreads of participants across conditions in the 3-way or higher order interactions involving metaphorical framing, metaphorical support and memory as predictors. These analyses are thus not reported upon.

#### Experiment 4

A logistic regression showed that both memory for the framing words and memory for the framing concepts were significantly influenced by metaphorical frame (words: *df* = 2, Wald  = 8.38, *p*<.05; concepts: *df* = 2, Wald  = 33.35, *p*<.001), but not by metaphorical support (words: *B = *−.17, *SE* = .13, *p* = .19; concepts: *B = *−.15, *SE* = .14, *p* = .25). The beast frame was significantly less well remembered than the non-metaphorical problem frame (words: *B* = −.34, *SE* = .16, *p*<.05; concepts: *B* = −.50, *SE* = .17, *p*<.001), whereas memory for the framing concept virus frame was better than memory for the non-metaphorical framing concept problem (*B* = .52, *SE* = .19, *p*<.01). Memory for the framing words “virus” and “problem” did not differ (*B* = .10, *SE* = .17, *p* = .54). Including the interaction did not improve model fit and is thus not reported upon. Including the interaction of metaphorical framing and metaphorical support makes that the main effect of metaphorical support becomes significant for both dependent variables (words: *B = *−.49, *p*<.05; concepts: *B = *−.56, *p*<.05), suggesting that participants remembered the framing words and concepts less well in the text with metaphorical support compared to the text without metaphorical support. However, model parameters showed that, for both dependent variables, including the interaction did not improve model fit compared to the model with only main effects.

Then, we conducted a 3 (metaphorical frame: beast, virus, no metaphor) ×2 (metaphorical support: present, absent) ×2 (memory for frame: correct, incorrect) ANOVA, with metaphorical frame, metaphorical support and memory for metaphorical framing word as between-subjects variables and policy preference as the dependent variable. As in our previous main analysis, there were no effects of metaphorical frame (*F*(2, 1014) <1), memory for metaphorical framing word (*F*(1, 1014) <1), or metaphorical support (*F*(1, 1014)  = 1.58, *p* = .21). All two-way interactions and the three-way interaction were also non-significant. Broadening our view of memory for framing word to memory for framing concept again yielded similar results.

Summarizing, all four experiments showed that there was no effect of memory for metaphorical frame, measured either as a framing word or a framing concept, on the overall policy preference findings. There was no interaction between metaphorical frame and memory for metaphorical frame either. We did find some effect of the presence of metaphorical support on memory for metaphorical frame in two of our four experiments: in Experiment 1, the virus frame was remembered better when it was accompanied by metaphorical support; in Experiment 2, this held for both the virus and the beast frame. This is in line with our view of the positive role of metaphorical support for the function of the metaphorical frame.

## Discussion

In this paper, we have reported four studies that comprise a follow-up study to Thibodeau and Boroditsky [1]. In contrast to the original studies, we consistently found no effects of metaphorical frames on policy preference. Additionally, there was no difference between the two metaphorical frames on the one hand and the non-metaphorical, neutral frame on the other hand, either. All three frames worked in the same way, consistently guiding all participants to a preference for enforcement-oriented policies. Our prediction that there might be an effect of metaphorical support for the metaphorical framing effects reported by Thibodeau and Boroditsky [1] was not supported either.

Across our four experiments, we tried to rule out alternative explanations for the differences between our findings and the original Thibodeau and Boroditsky [1] studies. In Experiment 1, we collected Dutch-language data in the Netherlands. To rule out cultural or linguistic differences, we translated our materials and questionnaire back into English and collected data in the US in Experiment 2, using the same online panel as used in the original Thibodeau and Boroditsky [1] studies (MTurk). In Experiment 3, we again collected US data from MTurk and removed our pre-test questions to rule out priming effects. We also re-analyzed our data using the same statistical procedure as employed by Thibodeau and Boroditsky ([1], Experiments 2-4). In order to check the possibility that the effect reported by Thibodeau and Boroditsky does not have a large or medium size but is a small effect, we collected a larger sample of>1,000 participants in Experiment 4 to have sufficient power to search for small effects. All of these analyses reveal similar results, in that we find no effects of metaphorical framing on reasoning. Instead, our only effect is a main effect of exposure that is irrespective of framing and irrespective of the presence or absence of metaphorical support for metaphorical framing.

Experiments 1 and 2 included a pre-test and demonstrated an effect of exposure, indicating that reading a text about crime makes people more likely to prefer an enforcement-oriented policy response (regardless of metaphorical frame). These findings tie in with studies indicating that increased media attention for crime-related topics in both fiction and non-fiction media can increase media consumers’ fear of crime and violence [18]–[21], make them perceive crime more as an important social problem [22], and influence support for crime-reducing policies [23]. Our results suggest a similar explanation, because participants favor enforcement-oriented, strong responses more after than before exposure to the text about crime.

An alternative explanation for the general preference for enforcement, for which we thank one of our reviewers, could lie in the construction of the particular stimulus text used as well as in the dependent variable. The current text presents the crime issue in terms of a crime outburst, in which cases enforcement may be preferable to reform. A text considering long-term crime prevention, in contrast, may lead to a general preference for reform. Similarly, our experiments mirror those of Thibodeau and Boroditsky ([1], Experiments 3–4) in presenting participants with five policy alternatives, three of which are valenced towards enforcement. It would perhaps be better to use a balanced set of alternatives (i.e., using an equal amount of enforcement and reform-oriented policy solutions). Future research using stimulus texts without such an implicit content bias (or, in contrast, an implicit content bias pointed towards reform) and a balanced set of policy options could help in unravelling these issues.

While we found no effects of metaphorical frame on reasoning, we did find that the metaphorical frames were remembered differently. First, in Experiment 2, the framing words “beast” and “virus” were remembered less well than the non-metaphorical ”problem”, while the “beast” concept was less well remembered than the “problem” concept, too; and in Experiments 3 and 4, “beast” was remembered less well than ”problem”. This may of course be partly due to the way of measurement, since the gap filling exercise “Crime is a …” prompts the word and concept “problem” as a default solution more than anything else. An exception to this pattern was found in Experiment 4, where the framing concept “virus” was remembered better than the framing concept “problem”. So, overall, these findings suggest that metaphorical frames like “beast” and “virus” do not always surpass such a non-metaphorical frame in terms of prominence or attention.

More interestingly, we found an interaction of metaphorical frame and metaphorical support on memory in two out of four experiments. In Experiment 1, memory for metaphorical concept “virus” was improved in the metaphorical support condition in comparison with the text without metaphorical support; in Experiment 2, memory for both “beast” and “virus” as framing words was improved in the texts with metaphorical support, and this also held for the “beast” framing concept. This is in accordance with our prediction, which held that metaphorical support increases activation of the metaphorical frame which in turn raises the chance of its being retrospectively remembered.

At the same time, however, none of these effects of metaphorical support on memory for metaphorical frame appeared to influence policy preference; nor did any differences between memory for metaphorical frame themselves influence policy preference. This is a finding that is identical with Thibodeau and Boroditsky [1] and suggests that retrospective awareness of metaphor is not related to framing effects of metaphor. It is of course possible that retrospective awareness of a word or concept in a text, however crucial that word or concept is, is not a good measure of how that word or concept worked during ambient processing, but that is a matter for further research.

The fact that our results do not correspond with the results of Thibodeau and Boroditsky [1], [9] suggests the need for establishing more precise boundary conditions under which metaphors do or do not impact reasoning. The literature contains some fruitful suggestions as to possible boundary conditions. First, many metaphor scholars [24]–[26] have argued that metaphors are not one homogeneous category, but that they can vary on several dimensions like novelty, artful deviation and deliberateness. These scholars suggest that variations of metaphors along these dimensions can influence their impact on recipients. This was in fact the original motivation of our present study, since both *A is B* metaphors (like “Crime is a beast/virus”) and extended metaphors (like, perhaps, the metaphorical support) can be seen as deliberate metaphors that can enhance metaphorical processing [25]–[26]. That such processes did not seem to occur in our present experiments may be due to a number of other factors.

For instance, the original study only included a predictor (metaphorical frame) and an outcome variable (policy preference). Future research should study potential mediators and moderators to present a more nuanced picture. The framing literature suggests potential moderators and mediators: people can for instance be influenced by frames if they have low political knowledge [27], or, conversely, when they display some degree of political knowledge [28]. For metaphorical frames, similar differences are observed: metaphorical frames are seen as more persuasive when they refer to self-relevant motives [29], but, conversely, also when readers experience a relatively large psychological distance, indicating that the topic is removed from them [30]. Similarly, while Thibodeau and Boroditsky [1], [9] suggest that metaphorical framing works unconsciously, other empirical evidence suggests that metaphors are more persuasive when they are actively recognized as metaphors [31]. Thus, it could be the case that our samples and those of Thibodeau and Boroditsky [1] differed along those lines.

In addition, some scholars suggest that metaphorical frames only have an effect when they are needed to understand the matter discussed in the text that follows (the “metaphor processing termination hypothesis” [32]). It might be the case that crime is a theme that participants in our experiment can easily understand (contrary, perhaps, to participants in Thibodeau and Boroditsky's experiments), and for which they thus do not need a metaphor to construct a clear image of the problem. In that case, framing crime as a beast or as a virus does indeed not necessarily matter and may not lead participants to change their opinions. To investigate for which themes metaphors possibly *do* influence people's opinions, we should change the topic of the stimulus text and test again. Thus, to firmly establish if and to generalize on how metaphors do or do not influence reasoning, similar results of metaphorical framing should be found using different texts and different policy metaphors [33].

A final point of note that may have incited our findings is that of timing in the sense of recent events. If fighting crime is high on the political or social agenda, people may already have an opinion about it. Some researchers have argued that we can only speak of a “‘true’ framing effect” [34] if this opinion is changed after presenting people with a certain frame [28].

However, if people do not already have an opinion about crime (which may sound unlikely, but it may be the case that crime problems are not really an issue for some people, for example because they live in a countryside village where crime is virtually non-existent and is not part of the political agenda), it is difficult to measure whether the frame actually changed a pre-existing belief. For topics that are remote and unimportant to readers, framing can also help to create a new belief rather than changing an existing belief [34]. Furthermore, framing may not only influence the content of belief, but also their strength or that the frame positively or negatively affected attitude strength. In comparing experiments with pre-test measures and experiments without pre-test measures, we can tease out these elements.

In all, we question Thibodeau and Boroditsky's [1] conclusion that natural language metaphors influence our reasoning. Our research has led to the conclusion that this issue should be rephrased as a question about the conditions under which metaphors do or do not influence our reasoning. These conditions do not only concern variation between metaphors and participants, but also the structure and function of the overall reading process in relation to prior beliefs, attitudes and intentions. By focusing on such boundary conditions, we will hopefully get a clearer picture of which metaphorical frames influence which types of people under which conditions.
